# Supplementary material for: Assessing Coagulation Parameters in Healthy Asian Elephants (Elephas maximus) from European and Thai Populations
Source: Animals (Basel). 2022 Feb 2;12(3):361. doi: 10.3390/ani12030361 (PMC8833339; doi:10.3390/ani12030361)
Supplement: Supplementary file 1 [file animals-12-00361-s001.zip › animals-1512129-supplementary Table S2_31012022.pdf]

Table S2. Positions of the SNPs detected in the F7 gene of Asian elephants from Thailand and from European zoos.

```
ATG GCT TCC CAT TCC CGC GGG CTC GCC CTT CTC TGC TTT CTG CTC   45
Met Ala Ser His Ser Arg Gly Leu Ala Leu Leu Cys Phe Leu Leu   15

GGG TTT CAG CAC CCT CTG ACA GCA GTC TTC ATG AAC CAG GAG GAA   90
Gly Phe Gln His Pro Leu Thr Ala Val Phe Met Asn Gln Glu Glu   30

GCC AAC AGC GTC TTA CAC AGG CAA AGG CGA GCC AAC AGT TTC TTC   135
Ala Asn Ser Val Leu His Arg Gln Arg Arg Ala Asn Ser Phe Phe   45

        G
GAA GAA CTG AGG TCA GGG TCA CTG GAG AGA GAG TGC AAG GAA GAA   180
Glu Glu Leu Arg Ser Gly Ser Leu Glu Arg Glu Cys Lys Glu Glu   60
        VAL
                             *
CAG TGC TCG TTC GAG GAA GCC AGG GAG ATC TTC AAG AGC ACT GAG   225
Gln Cys Ser Phe Glu Glu Ala Arg Glu Ile Phe Lys Ser Thr Glu   75

AGG ACT AGG CAG TTC TGG GTG GCT TAT ACC GAT GGA AAC CAG TGC   270
Arg Thr Arg Gln Phe Trp Val Ala Tyr Thr Asp Gly Asn Gln Cys   90

             T
ACC TCA AAC CCG TGC CAG AAT GGG GGC CTG TGT GTG GAC CAG CTC   315
Thr Ser Asn Pro Cys Gln Asn Gly Gly Leu Cys Val Asp Gln Leu   105
            Leu

CAG TCT TAC ATT TGC TTC TGC CTT GAT GAT TTT GAG GGT CGG AAC   360
Gln Ser Tyr Ile Cys Phe Cys Leu Asp Asp Phe Glu Gly Arg Asn   120

                                 A
TGT GAG ACA AAC AAA AAC AGC CAG CTG ATC TGT CTG AAT GAA AAC   405
Cys Glu Thr Asn Lys Asn Ser Gln Leu Ile Cys Leu Asn Glu Asn   135
                                Gln

GGA GGC TGT GAA CAG TAC TGC AGT GAC AAC GCA GAG ACC AAG CGT   450
Gly Gly Cys Glu Gln Tyr Cys Ser Asp Asn Ala Glu Thr Lys Arg   150

         A
TCC TGC CGA TGT CAT GAC GGC TAC ACG CTC ATG GCT GAT GGA GTG   495
Ser Cys Arg Cys His Asp Gly Tyr Thr Leu Met Ala Asp Gly Val   165
        Gln

TCC TGC ACG CCC ACA GTT GAA TAT CCG TGT GGA AAA ATA CCT GTT   540
Ser Cys Thr Pro Thr Val Glu Tyr Pro Cys Gly Lys Ile Pro Val   180

CTG GAA AAA AGA AAT GAC AAC ATC CCC CAA GGC CGA ATT GTG GGT   585
Leu Glu Lys Arg Asn Asp Asn Ile Pro Gln Gly Arg Ile Val Gly   195

GGC AGG TTG TGT CCC AAA GGG GAG TGT CCA TGG CAG GCT GTG ATA   630
Gly Arg Leu Cys Pro Lys Gly Glu Cys Pro Trp Gln Ala Val Ile   210

AAG CTG CAG GGG ACT CTG CTG TGT GGG GGA TCT CTG CTT GAC GCC   675
Lys Leu Gln Gly Thr Leu Leu Cys Gly Gly Ser Leu Leu Asp Ala   225

ACC TGG GTG GTC TCC GCA GCC CAC TGT TTC AAC AAA CCC GGC ATC   720
Thr Trp Val Val Ser Ala Ala His Cys Phe Asn Lys Pro Gly Ile   240

CTC AGG AAC TGG GAG AAT ATA ACA GTG GTG TTG GGT GAG CAC GAC   765
Leu Arg Asn Trp Glu Asn Ile Thr Val Val Leu Gly Glu His Asp   255

TTT AGT GAC GAG GAC GGC GAT GAA CAA GAA CGG CGA ATT GCT CAG   810
Phe Ser Asp Glu Asp Gly Asp Glu Gln Glu Arg Arg Ile Ala Gln   270

ATC ATA ATC CCT GAC AAG TAT GTG TCA GGC AAG ACC GAC CAC GAC   855
Ile Ile Ile Pro Asp Lys Tyr Val Ser Gly Lys Thr Asp His Asp   285

ATT GCC CTG CTG CGC CTG AGA ACG CCG GTG AAC TTC ACT GAC TAC   900
```

Ile Ala Leu Leu **Arg** Leu Arg Thr Pro Val Asn Phe Thr Asp Tyr 300

GTA GTG CCC CTC TGT TTG CCT GAC AAG AGA TTC TCA GAG CAA ACA 945
Val Val Pro Leu Cys Leu Pro Asp Lys Arg Phe Ser Glu Gln Thr 315

CTC GCC TTC ATC CGT TTC TCC TCC GTG **AGC** GGC TGG GGC CAG CTT 990
Leu Ala Phe Ile Arg Phe Ser Ser Val **Ser** Gly Trp Gly Gln Leu 330

CTC GAC AGG GGC GCC ACA GCC CTC GAG CTC ATG ACT ATA GAC GTG 1035
Leu Asp Arg Gly Ala Thr Ala Leu Glu Leu Met Thr Ile Asp Val 345

CCC AGG CTG ATG ACC CAG GAC TGT AAT GAG CAA ATG CAA AGG ACC 1080
Pro Arg Leu Met Thr Gln Asp Cys Asn Glu Gln Met Gln Arg Thr 360

GCC AAC TCC CCA GTG GTG ACC GAG AAC ATG TTC TGT GCT GGC TAC 1125
Ala Asn Ser Pro Val Val Thr Glu Asn Met Phe Cys Ala Gly Tyr 375

CTG GAT GGG ACC AAG GAT GCC TGC AAG GGT GAC **AGT** GGG GGC CCT 1170
Leu Asp Gly Thr Lys Asp Ala Cys Lys Gly Asp **Ser** Gly Gly Pro 390

CAT GCC ACC AAG TAC CGA AAC ACA TGG TAC CTG ACA GGA ATT GTC 1215
His Ala Thr Lys Tyr Arg Asn Thr Trp Tyr Leu Thr Gly Ile Val 405

AGC TGG GGT GAG GGC TGT GCA GCC GTG GGC CAC GTT GGG GTG TAC 1260
Ser Trp Gly Glu Gly Cys Ala Ala Val Gly His Val Gly Val Tyr 420

ACC AGG GTC TCC CGG TAC ATT GAG TGG CTG AAC AGG CTC ATG GAC 1305
Thr Arg Val Ser Arg Tyr Ile Glu Trp Leu Asn Arg Leu Met Asp 435

TCG AAC CCG AGC CCA GGC CGT TTC CTG TCA GCC CGT TTT CCC TAG 1350
Ser Asn Pro Ser Pro Gly Arg Phe Leu Ser Ala Arg Phe Pro End 450

Triplets containing SNPs are labelled in bold together with the amino acid coded by that triplet. The actual position of the SNP is underlined. Grey boxes indicate SNPs for which one allele is causing a missense (non-synonymous) mutation. Nucleotides of the alternative allele are given on top of the respective SNP, amino acid changes are indicated below the SNP. Numbers at the right indicate position of the last nucleotide (top line) or the last amino acid (lower line). Nucleotide and amino acid positions are based on the African elephant (*Loxodonta africana*) cDNA of the F7 gene coding for coagulation factor VII (without 5′ untranslated region). * Previously reported mutation found in Asian elephants [11], but not present in the our European and Thai elephant study population.
